# The Role of Mitochondrial DNA Mutations in Cardiovascular Diseases

**DOI:** 10.3390/ijms23020952

**Published:** 2022-01-16

**Authors:** Siarhei A. Dabravolski, Victoria A. Khotina, Vasily N. Sukhorukov, Vladislav A. Kalmykov, Liudmila M. Mikhaleva, Alexander N. Orekhov

**Affiliations:** 1 Department of Clinical Diagnostics, Vitebsk State Academy of Veterinary Medicine (UO VGAVM), 7/11 Dovatora Str., 210026 Vitebsk, Belarus; 2 Laboratory of Cellular and Molecular Pathology of Cardiovascular System, AP Avtsyn Research Institute of Human Morphology, 3 Tsyurupa Street, 117418 Moscow, Russia; nafany905@gmail.com (V.A.K.); vasily.sukhorukov@morfolhum.ru (V.N.S.); 3 Laboratory of Angiopathology, The Institute of General Pathology and Pathophysiology, 8 Baltiyskaya Street, 125315 Moscow, Russia; xxor2011@gmail.com; 4 Laboratory of Medical Genetics, Russian Medical Research Center of Cardiology, Institute of Experimental Cardiology, 15-a 3-rd Cherepkovskaya Str., 121552 Moscow, Russia; 5 AP Avtsyn Research Institute of Human Morphology, 3 Tsyurupa Street, 117418 Moscow, Russia; mikhalevalm@yandex.ru; 6 Institute for Atherosclerosis Research, Osennyaya Street 4-1-207, 121609 Moscow, Russia; a.h.opexob@gmail.com

**Keywords:** cardiovascular diseases, atherosclerosis, mitochondria, coronary artery disease, Brugada syndrome, hypertension, ischemic stroke

## Abstract

Cardiovascular diseases (CVD) are one of the leading causes of morbidity and mortality worldwide. mtDNA (mitochondrial DNA) mutations are known to participate in the development and progression of some CVD. Moreover, specific types of mitochondria-mediated CVD have been discovered, such as MIEH (maternally inherited essential hypertension) and maternally inherited CHD (coronary heart disease). Maternally inherited mitochondrial CVD is caused by certain mutations in the mtDNA, which encode structural mitochondrial proteins and mitochondrial tRNA. In this review, we focus on recently identified mtDNA mutations associated with CVD (coronary artery disease and hypertension). Additionally, new data suggest the role of mtDNA mutations in Brugada syndrome and ischemic stroke, which before were considered only as a result of mutations in nuclear genes. Moreover, we discuss the molecular mechanisms of mtDNA involvement in the development of the disease.

## 1. Introduction

Atherosclerosis is a chronic inflammatory disease of large and medium-sized arteries that causes other dangerous complications, which are collectively defined as CVD (cardiovascular diseases). Atherosclerosis is characterized by the accumulated deposition of lipoproteins and the migration of monocyte and macrophage artery walls, leading to the restriction of circulation and the risk of thrombosis. Despite the continuing investigation for atherosclerosis’s mechanisms and causes, as well as the search for novel drugs and means for improving healthcare and quality of life, atherosclerosis and the subsequent CVD are consistently high around the globe. Currently, atherosclerosis is an important socio-economic problem and is the leading cause of death worldwide [1].

Coronary artery disease (CAD, also known as atherosclerotic heart disease) is the most common heart disease, and is caused by stenosis in coronary arteries and their branches. During the classical genetics period, candidate genes and polymorphism sites were selected for analysis based on our knowledge of the disease’s pathophysiology. Due to the low-throughput technologies and a low number of subjects, many false positive (and false negative) results were common for this period. The next period, with the GWAS (genome-wide association study), has entailed a significant improvement and extension of our knowledge of the genetic background and pathogenesis of many diseases [2]. The application of GWAS to search for CAD risk alleles has led to the identification of new genes and many significant variants, which greatly expand our understanding of the disease, with a total of 164 chromosomal loci identified [3]. Accumulating evidence supports the idea that genetic factors play a crucial role in atherosclerosis pathogenesis; however, the exact underlying mechanisms are still not fully understood. Nevertheless, the analysis of family history and screening for verified gene variants are effective strategies to predict the individual risk of atherosclerosis development [4].

In addition to nuclear gene variants, mitochondrial mutations also play a crucial role in many human diseases, including atherosclerosis and other CVD [5]. The mitochondria produce ATP (adenosine triphosphate) and harmful ROS (reactive oxygen species) as by-products, which are normally effectively neutralized by antioxidants. However, mutated mitochondrial genes unbalance cellular respiration and energy production, leading to extreme ROS production, oxidative damage and mitochondria dysfunction, further triggering cellular damage, apoptosis and cell death [6]. Mitochondria dysfunction is the first step in atherosclerosis development: an altered ROS (reactive oxygen species) level causes vascular EC (endothelial cell) dysfunction and the recruitment of circulating immune cells, initiating immune reactions for further atherosclerotic plaque formation. Thus, mitochondria represent a promising target for pharmaceutical intervention [7]. The pathogenesis of atherosclerosis starts from local endothelial dysfunction and the inflammatory response of the arterial wall. Further, the emerging atherosclerotic lesion site expresses adhesion molecules, lipoproteins (LDL (low-density lipoprotein) is the main fraction) penetrate the arterial wall and cause local accumulation, subsequently leading to plaque formation [8,9].

In this review, we discuss the recently identified mutations in mitochondrial DNA associated with CVD (CAD, hypertension, ischemic stroke and Brugada syndrome), their significance as diagnostic and therapeutic targets. Additionally, molecular mechanisms, linking mtDNA mutations and CVD development and progression are discussed.

## 2. Mitochondrial Genome Organization, Functions and Dynamics

The mitochondrial genome is represented by a circular double-stranded mtDNA with an approximate length of around 16.5 thousand nucleotides. The mitochondrial genome encodes only 37 genes (13 structural genes encoding subunits of OXPHOS (oxidative phosphorylation complexes)), 22 tRNAs (transport RNAs) and 2 rRNAs (ribosomal RNAs). All other proteins necessary for mitochondrial function, transcription, repair and maintenance, are encoded by a nuclear genome and imported to the mitochondria [10]. Because every organelle contains a different number of mtDNA copies, occurring mutations could be homoplasmic (all mtDNA copies are identical and carry this particular mutation) or heteroplasmic (only some mtDNA copies have the mutation) [11]. Mitochondrial mutations are often associated with human diseases, and they could be inherited from the mother or acquired and accumulated during the individual’s life [12]. The effect of mitochondrial mutations depends on the affected tissue and the heteroplasmy level [13]. A mutant-specific phenotype develops when a certain threshold of mutation heteroplasmy is reached [11]. Because mtDNA mutations are normally masked by functional wild-type copies, the threshold of heteroplasmy is usually high (more than 70%); however, this value could be tissue specific [14].

The most crucial role of the mitochondria is energy production in the form of ATP. There are several steps in ATP synthesis: (1) the conversion of pyruvate and fatty acids into acetyl-CoA; (2) the Krebs cycle uses acetyl-CoA to produce NADH (nicotine amide adenine dinucleotide); (3) electron transfer from NADH to oxygen via the respiratory chain; and (4) ATP synthesis by the membrane ATP synthase [15]. ATP synthesis is accompanied by ROS production, which has some regulatory and signalling roles [16]. However, excessive ROS generation could lead to oxidative damage to biomolecules (mtDNA, proteins, lipids) [17]. Nowadays, excessive ROS and subsequent oxidative damage are recognized as two of the main factors of atherosclerosis pathogenesis [6].

Mitochondria participate in the regulation of Ca^2+^ homeostasis via their close interaction with ER (endoplasmic reticulum), the main cellular Ca^2+^ reservoir. The mechanisms of Ca^2+^ flux in/from the mitochondria is crucial for cellular signalling, neurotransmitter and hormone release, the regulation of mitochondrial membrane potential and respiratory bioenergetics [18]. Additionally, disturbance in the mitochondrial lipid metabolism could activate ER stress and UPR (unfolded protein response) via mitochondria–ER contact sites [19].

The processes of mitochondrial biogenesis, turnover and recycling are directed based on the equal distribution of mitochondria between dividing cells, the maintenance of the healthy and efficient mitochondrial population and the salvage of damaged or ineffective mitochondria, respectively. Mitochondrial turnover consists of cycles of fission (split) and fusion (merge) of dysfunctional or damaged organelles. Separated dysfunctional parts of the mitochondria are further degraded via a specialized form of autophagy—mitophagy. Healthy parts of the mitochondria are fused and continue normal functioning as a part of the mitochondrial network [20].

*DNM1L* (dynamin 1 like) and *FIS1* (fission, mitochondrial 1) are the main genes responsible for mitochondrial fission, and *MFN1*, *MFN2* (mitofusin 1 and 2) and *OPA1* (optic atrophy protein 1) are responsible for fusion. The expression of those proteins and the activity of the corresponding proteins are tightly regulated on several levels. Their excessive or inadequate expression/activity would lead to impaired mitophagy, which was shown to be associated with many human diseases [21], including CVDs [22]. However, the importance of mitochondrial turnover mechanisms also suggests their great potential as a therapeutic target. Recently, a mitochondrial fission inhibitor, mdivi1, was used on the mice Drp1^+/−^ model of AAA (abdominal aortic aneurysm), where it helped to prove the key role of mitochondrial fission in AAA development [23].

Mutations in the nuclear genes that encode the respiratory chain subunits and proteins responsible for mtDNA maintenance (replication, transcription and copy number control), biogenesis and dynamics (fission and fusion) could cause the secondary instability of the mitochondrial genome and mitochondria dysfunction. Mitochondrial fragmentation and cardiomyopathy are caused by mutations in *OPA1* (responsible for fusion), *SLC25A4* (mitochondrial solute carrier family 25 member 4) (responsible for ADP/ATP balance and mtDNA stability) and *DRP1* (responsible for fission) (reviewed in [24,25]). Similarly, cardiomyopathies were described for the nuclear encoded genes required for proper OXPHOS forming, including CI assembly factors (*NDUFAF1*, *ACAD9*), CIII assembly factors (*UQCC3*), CIV assembly factors (*COA5*), CV assembly factors (*TMEM70*) and other genes (reviewed in [26]).

Mitochondrial diseases are the focus of many researchers. While there are still many unanswered questions, some pioneering treatments and preventive methods are already available [27]. Some major strategies can be defined: (1) enhance the efficacy of the electron transfer chain (with the application of thiamine, coenzyme Q_10_, idebenone), improve the energy buffer (creatine), cardiolipin protection (elamipretide), NO production (with amino acids arginine and citrulline), antioxidants supplementation (vitamin E, D, C) and others [28]. Additionally, mitochondria transplantation, mitochondrial gene therapies [29] and the application of pharmacological agents to modulate mitochondrial metabolism and functions are under intensive investigation [30].

## 3. Mitochondrial DNA Mutations

The mutation rate of mtDNA is much higher in comparison to nuclear DNA. There are several mechanisms leading to such an outcome. Firstly, ROS damage is currently recognized as the major driver of mtDNA mutagenesis. Further mutation accumulation leads to more severe mitochondrial dysfunction and even higher ROS production, thus forming a vicious circle [31]. Secondly, the mitochondrial DNA repair system is not so efficient in comparison to the nuclear one [32]. Additionally, mtDNA is not protected by histone proteins, and thus is more accessible for harmful agents. Finally, mutations in polymerase γ (POLG), which is responsible for the replication of mtDNA, is the most common cause of inherited mitochondrial disorders [33].

Further in this section, we analyse the recently identified mtDNA mutations associated with CVDs (CAD (Table 1), hypertension (Table 2), ischemic stroke and Brugada syndrome (Table 3)).

The mtDNA mutations associated with CAD could be grouped into several main categories, each with different mechanisms of action: (1) mutations in tRNA were predicted to destabilize the base pairing at the affected sites, potentially altering the secondary structure of this tRNA and causing its quicker degradation and the subsequent reduction in mitochondrial protein levels [41]; (2) mutations in the OXPHOS components were shown to reduce ATP synthesis and increase ROS production; and (3) mutations in the D-loop would interrupt the normal mtDNA replication process, resulting in the reduced mtDNA copy number [38]. Interestingly, the majority of identified mtDNA mutations were found to be non-pathogenic or mild; thus, they may not be able to cause a certain pathogenic phenotype development [42]. However, the disease could manifest under the influence of other genetic, nutritional or environmental factors [40].

Hypertension (high blood pressure) is a common public health problem, affecting more than 1.28 billion people worldwide. Hypertension (HTN) can be primary (essential, with no identifiable cause and developing over many years) and secondary, which is caused by various other conditions (kidney, lung diseases, thyroid problems, sleep apnoea and others). HTN is a risk factor for CAD, stroke, heart failure and renal dysfunction [43].

Essential HTN is known as a multifactorial disease, where both environmental and genetic factors are responsible for the physiopathology and severity of the disease manifestation. The role of genetic factors in HTN has a long history being investigated, with studies attributing a tendency to familial aggregation to this disease despite different environmental factors [44]. Recently, several studies have identified multiple mtDNA mutations associated with HTN, thus suggesting its maternal transmission [45,46,47]. A wide-scope study, conducted in 2007, has defined the fraction of mitochondria-mediated cases among hypertensive pedigrees as 35.2% [48]. Interesting, in north China, the number of HNT incidences is much higher than average [49]. Unsurprisingly, the majority of current investigations have been conducted in this ethnic group (Chinese, Chinese Han and Mongolian Chinese) (Table 2); however, in other ethnic groups, HTN incidence may have a different rate and fraction of mitochondria-mediated maternal transmission.

**Table 2:** List of mtDNA mutations associated with hypertension.

Mutation	Gene	Other Notes	References
Mt3970 (C > T)	MT-ND1	Chinese MIEH patients	[50]
Mt4048 (G > A)			
Mt4071 (C > T)			
Mt4086 (C > T)			
Mt4164 (A > G)			
Mt4248 (T > C)			
Mt4386 (T > C)	tRNA^Gln^		
Mt4394 (C > T)			
Mt8414 (C > T)	MT-ATP8		[51]
Mt8701 (A > G)	MT-ATP6		
Mt8584 (G > A)			
Mt8273_8281del			
Mt8701 (A > G)	MT-ATP6	A Chinese family with MIEH cases	[52]
Mt5587 (T > C)	tRNA^Ala^		[53]
Mt12280 (A > G)	tRNA^Leu(CUN)^		
Mt5512 (A > G)	tRNA^Trp^		[54]
Mt15077 (G > A)	MT-CYB		[55]
Mt15992 (A > G)	tRNA^Pro^		
Mt10410 (T > C)	tRNA^Arg^		[56]
Mt10454 (T > C)			
Mt3253 (T > C)	tRNA^Leu(UUR)^	Chinese Han EH patients	[57]
Mt15910 (C > T)	tRNA^Thr^		[58]
Mt5655 (T > C)	tRNA^Ala^	Han Chinese family with EH	[59]
Mt4401 (A > G)	Between tRNA^Met^ and tRNA^Gln^		
Mt7471 delC	tRNA^Ser(UCN)^		[60]
Mt4467 (C > A)	tRNA^Met^		[61]
Mt4263 (A > G)	tRNA^Ile^		[62]
Mt15909 (A > G)	tRNA^Thr^		[63]
Mt4363 (T > C)	tRNA^Gln^		[64]
Mt5601 (C > T)	tRNA^Ala^		[65]
Mt4435 (A > G)	tRNA^Met^		

Brugada syndrome (BrS), described in 1992 by the Brugada brothers, is a rate cardiac disorder characterized by a structurally normal heart, but with typical electrocardiogram alterations and a high risk of sudden death. BrS accounts for 4% of all sudden deaths and 20% of sudden deaths in the absence of structural heart disease [66]. Approximately 80% of Brugada patients are male of age 40–45, and symptoms develop during night or daytime rest periods and are often combined with fever. Diagnosis is based on the characteristic electrocardiogram pattern with a cove-shaped ST elevation in leads V1 to V3. The most effective strategy to prevent sudden cardiac death is the application of an implantable cardioverter-defibrillator. However, this approach has many drawbacks for the patient [67].

BrS is a genetically transmitted disease with an autosomal dominant transmission and incomplete penetrance. The *SCN5A* (sodium voltage-gated channel alpha subunit 5) gene, which encodes the α-subunit of the Na+ channel, is considered to be the main genetic factor responsible for BrS. Mutations in *SCN5A* are found in 25–30% of BrS patients, with over 300 mutations known for the *SCN5A* gene being associated with BrS [68,69]. In addition to *SCN5A*, mutations in 17 other genes are also known to be responsible for a small number of Brugada cases [70], suggesting the co-segregation of different involvement mutations or genetic variants in the clinical manifestation of this disorder.

BrS is considered endemic in Southeast Asian countries, where the number of cases is higher in comparison to the average number (5–20 cases for every 10,000 people worldwide) [71]. There are only two recent reports suggesting the involvement of mtDNA mutations in BrS (Table 3) [72,73] (Table 3). Taking into account that the current genetic monitoring covers only 30% of BrS patients, the identification of additional biomarkers associated with BrS could be particularly beneficial for the early diagnosis of asymptomatic patients.

Ischemic stroke (IS) is a multifactorial disorder characterized by the sudden loss of blood circulation to an area of the brain due to cerebral artery stenosis or occlusion, resulting in brain ischemia, hypoxia or necrosis. Oxidative stress, energy disturbance, excitatory amino acid toxicity, neuroinflammation and nerve cell death are caused by brain ischemia and form a complex network, leading to subsequent cascade damage [74]. Mitochondrial dysfunction is known to be involved in neuronal cell death and oxidative damage in neurodegenerative and CVD via the triggering of several molecular mechanisms, leading to vascular dysfunction [75]. The identified MtDNA variants could be used as diagnostic and IS-predicting biomarkers. Similarly, identified IS-protective MtDNA mutations (Table 3) could be used in future research to better understand the aetiology of IS.

**Table 3:** List of mtDNA mutations associated with Brugada syndrome and ischemic stroke.

Mutation	Gene	Other Notes	References
Mt4216 (T > C)	MT-ND1	Associated with the most severe BrS phenotype among Caucasian BrS patients	[72]
Mt11251 (A > G)	MT-ND4		
Mt15452 (C > A)	MT-CYB		
Mt16126 (T > C)	D-loop		
Mt4377 (T > A)	tRNA^Gln^	Associated with BrS in Iranian patients	[73]
Mt4407 (G > A)	tRNA^Met^		
Mt4456 (C > T)			
Mt5580 (T > C)	junction region between tRNA^Trp^ and tRNA^Ala^		
m.16145G > A	D-loop	Genetic risk factors for IS	[76]
m.16311T > C			
Mt195 (T > C)	D-loop	Protective factors of IS in Chinese patient cohort	[77]
Mt311 (C > T)	D-loop		
Mt12338 (T > C)	MT-ND5		

## 4. Molecular Mechanisms of mtDNA Mutations

**Figure 1:**
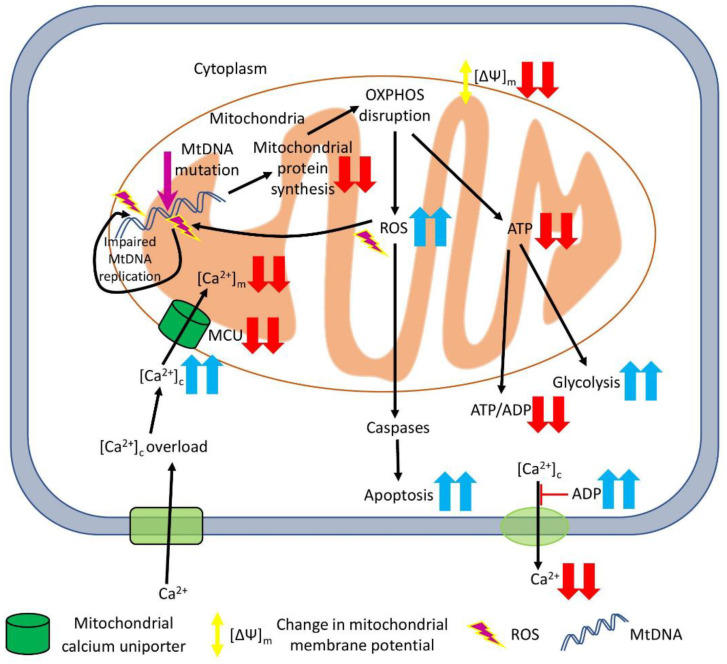
Potential pathways affected by mtDNA mutations and associated with CVD. The mtDNA mutations lead to a decreased level of tRNAs and protein synthesis (including proteins involved in mitochondrial OXPHOS), and increased ROS production and, subsequently, mitochondrial oxidative stress and cell apoptosis. High ROS levels tend to cause more mutations in mtDNA during its replication. OXPHOS malfunction leads to lower ATP output and the collapse of the mitochondrial membrane potential, thus changing the ATP/ADP ratio, interrupting normal ion traffic and stimulating glycolysis as an alternative ATP-generation pathway. An impaired Ca^2+^ metabolism can cause the accumulation of Ca^2+^ in the cytosol, with low concentrations in mitochondria and extracellular space, which may result in cell swelling and death. Red arrows represent decrease, blue – increase of a particular process/parameter.

The exact molecular mechanisms connecting mtDNA mutations with different CVD are not fully understood. However, multiple studies suggest that mtDNA mutations disrupt mitochondrial homeostasis, causing a rise in ROS production, dysregulating Ca^2+^ metabolism and reducing energy synthesis. The molecular pathways affected by mtDNA mutations are briefly summarised in Figure 1.

Dysfunctions of OXPHOS in the mitochondria lead to abnormal energy metabolism and abnormal changes in the exchange of sodium and calcium, leading to calcium overload in the cytoplasm, the diastolic dysfunction of cardiomyocytes and smooth muscle cells, and promoting blood pressure increase.

### 4.1. Mutations in tRNA Genes

tRNA genes are normally highly conserved due to their indispensable role in the biosynthesis of mitochondrial proteins. Thus, mutations in tRNA are predicted to decrease tRNA stability and destabilize the base pairing, potentially altering the secondary structure of this tRNA. Previously, those effects have been reported for the Mt10398 A > G mutation in the *MT-ND3* gene in hypertension-associated stage renal disease [78].

The cybrid cells carrying the Mt15910 (C > T) mutation (tRNA^Thr^) had 37.5% lower levels of tRNA^Thr^ in comparison to healthy control cells, suggesting that the Mt15910 (C > T) mutation speeds up its degradation [41]. Previously, similar results were described for other tRNA mutations, Mt3243 (A > G) tRNA^Leu(UUR)^ [79], Mt5655 (T > C) tRNA^Ala^, Mt10003 (T > C) tRNA^Gly^, Mt3253 (T > C) tRNA^Leu(UUR)^, Mt7551 (A > G) tRNA^Asp^ and Mt14692 (A > G) tRNA^Glu^ [45]. Subsequently, alterations of the tRNA levels lead to the reduced rate of mitochondrial protein synthesis and a reduction in mitochondrial protein levels in the mutant cells, altered complex I/III activity, electron leakage and a rise in ROS production [41]. Elevated levels of ROS production could lead to the damage of cellular biomolecules and contribute to the disease-related phenotype (discussed in the following section).

However, most likely is that, in addition to the mitochondrial mutations, other nuclear genetic or epigenetic, as well as a combination of environmental and lifestyle factors are responsible for the development of the clinical phenotype of the particular CVD. For example, platelet mitochondrial DNA methylation was proposed as a new biomarker to predict CVD [80,81]. As it was shown, mtDNA methylation could be affected by environmental stress factors (such as air pollution), causing mitochondrial dysfunction and, subsequently, affecting heart functions [82,83]. Similarly, mtDNA methylation could be influenced by a diet supplemented with l-carnitine and trimethylamine-N-oxide (TMAO), which are known to be CVD biomarkers. While the exact mechanism and role of mtDNA methylation are not fully understood, the high TMAO level was associated with a worse lipid profile and an increased risk of major adverse cardio and cerebrovascular events [84]. Dietary antioxidants could also remodel mtDNA methylation patterns and provide a positive effect on the initiation, development and progression of many chronic diseases (such as type 2 diabetes, Alzheimer’s disease, cancer and atherosclerosis) (reviewed in [85]). For more information, we wish to redirect interested readers to the following recent reviews [86,87].

### 4.2. Mitochondrial Oxidative Stress

Mitochondria are involved in the regulation of apoptosis, cell cycle and cell development, ROS production and cell signal transmission and intracellular Ca^2+^ homeostasis. However, the major function of mitochondria is energy generation (in the form of ATP), covering approximately 95% of cells’ active demands. This function is vital in energy-consuming cells, such as neurons and cardiomyocytes [88]. Thus, mitochondrial dysfunction and injury may have significant effects on general cell function. Several recent reports suggest that atherosclerosis progression and development are closely related to mitochondrial structure, abnormal function and, especially, energy metabolism. The application of anti-miR33 therapy (miR33 is a known repressor of several energy-metabolism responsible genes, namely, *PGC-1α* (PPARG coactivator 1 alpha), *PDK4* (pyruvate dehydrogenase kinase 4) and *SLC25A25* (calcium-binding mitochondrial carrier protein ScaMC-2)) resulted in enhanced mitochondrial respiration and ATP production, the subsequent stimulation of macrophage cholesterol efflux and the reduction of atherosclerosis [89]. Similarly, the application of CoQ10 (coenzyme Q10—one of the mitochondrial respiratory chain components) improved mitochondrial function, inhibited ROS production and enhanced energy metabolism, thus attenuating atherosclerosis [90].

ROS is one of the main factors contributing to oxidative stress in the body and promoting cardiovascular diseases. ROS is a normal cellular component, presenting usually at low concentrations, and is known to maintain vascular integrity by regulating endothelial function and vascular contraction–relaxation. However, under pathological conditions, the ROS levels rise dramatically and could damage vascular endothelial cells and cause endothelial dysfunction. An increase in ROS levels plays a key role in the pathogenesis of CVD through the proliferation and migration of VSMC (vascular smooth muscle cells), the stimulation of inflammation mediator release and an increase in free calcium in endothelial cells. In cardiomyocyte mitochondria, and increased ROS leads to the loss of mtDNA and increased autophagy [91]. Currently, ROS-targeted approaches are the most promising type of anti-atherosclerotic therapies [92].

Several mitochondria mutations have been studied in detail. The tRNA^Ile^ mutation at the position Mt4263 (A > G) is involved in malfunctioning respiratory complexes I, III and IV, which are significantly enriched in AUC and/or AUU codons that pair with tRNA^Ile^(GAU). Thus, the resulting oxygen consumption was reduced to 70−80% of normal levels [93]. The Mt15910 (C > T) mutation in tRNA^Thr^, known to be linked to CHD, on the organelle level was associated with a 37.5% reduction in tRNA^Thr^ levels and a 25% decrease in mitochondrial translation rates [41]. Further, a damaged mitochondrial respiratory chain leads to a vicious cycle: increased ROS production means a higher rate of mtDNA mutations and cell death.

### 4.3. Mitochondrial Energy Synthesis

mtDNA mutations and deletions disrupt the normal functioning of the respiration chain and decrease proton flow, thus reducing mitochondrial membrane potential and inhibiting mitochondrial ATP synthesis. A recent study showed that the Mt3253 (T > C) mutation (tRNA^Leu(UUR)^) resulted in the decreased activity of mitochondrial complexes I and V, leading to 66% lower ATP production, reduced membrane potential and the increased production of ROS [57]. Similarly, the Mt10454 (T > C) and Mt10410 (T > C) mutations significantly reduced mitochondrial ATP and membrane potential, increased ROS production and raised the levels of MDA (malondialdehyde) and 8-OhdG (8-Oxo-2’-deoxyguanosine), while levels of SOD (superoxide dismutase) and GSH-Px (glutathione peroxidase) were decreased [56]. Cell lines carrying the Mt4467 (C > A) mutation (tRNA^Met^) had decreased oxygen consumption, 26.2% lower mitochondrial membrane potential, 26.4% lower ATP level, and 114.5% higher ROS production [61]. The Mt15909 (A > G) mutation (tRNA^Thr^) resulted in the decrease in the overall levels of mitochondrial translation products and ATP production, while ROS generation was increased [63]. Therefore, MtDNA mutations and deletions contribute to oxidative stress and mitochondrial dysfunction, which may be involved in the development and pathogenesis of CVD, in particular, hypertension and atherosclerosis.

### 4.4. Mitochondrial Ca^2+^ Regulation

Mitochondria could affect cytosolic Ca^2+^ levels as a result of both mtDNA mutations and nuclear gene mutations. The direct pathway would affect Ca^2+^ uptake into the mitochondria via the MCU (mitochondrial calcium uniporter). Cells carrying the Mt4263 (A > G) mutation (tRNA^Ile^) exhibited a lower expression of MCU, which resulted in dysregulated Ca^2+^ uptake and cytoplasmic Ca^2+^ overload [62]. Various mutations in nuclear-encoded GARS (glycyl-tRNA synthetase 1) resulted in alterations to the mitochondrial respiratory chain complex subunits, Krebs cycle enzymes, assembly genes and the proteins involved in fatty acid oxidation, thus causing mitochondrial cardiomyopathy. Specifically, mitochondrial calcium metabolism and ER–mitochondria interactions sites were altered, which contributed to the clinical presentations of the inherited neuropathies [94]. Additionally, indirectly, Ca^2+^ transport depends on the available ATP. MtDNA mutations cause a decrease in ATP synthesis and mitochondrial membrane potential, which could lead to Ca^2+^ dysregulation, malfunction in smooth muscle and apoptosis [95].

The MCU plays a key role in the transport of Ca^2+^ between the mitochondria and the sarcoplasmic reticulum in skeletal muscle cells and cardiomyocytes [96]. The knock-out of myocardial cell mitophagy regulating protein BNIP3 (BCL2 interacting protein 3) leads to Ca^2+^ transport in the ER, mitochondrial injury, a rise in the apoptosis rate and left ventricular myocardial fibrosis [97]. FOXO3a (forkhead box O3a) upregulates *BNIP3* expression in normal and stressed cardiomyocytes, with subsequent increases in mitochondrial Ca^2+^, leading to decreased mitochondrial membrane potential, mitochondrial fragmentation and apoptosis. Therefore, FOXO3a/BNIP3-mediated Ca^2+^ regulation contributes to mitochondrial dysfunction in heart failure and could be used as a potential therapeutic target [98]. The upregulation of SERCA2a (sarcoendoplasmic reticulum calcium ATPase 2a) facilitated greater depolarization-induced Ca^2+^ transience and increased endoplasmic reticulum and mitochondria Ca^2+^ load in spontaneously hypertensive rats stellate neurons [99]. The application of MCU inhibitors was shown to inhibit excessive mitophagy in neurons in the ischemia/reperfusion model, thus suggesting an additional Ca^2+^-mediated protective mechanism [100].

In total, several molecular mechanisms are associated with mitochondrial Ca^2+^ homeostasis and the pathogenesis of CVD. The alteration of mitochondrial Ca^2+^ levels can affect mitochondrial autophagy, fission and fusion, mitochondrial morphology and function, and thus could be used as a target in CVD prevention and treatment.

### 4.5. MtDNA Copy Number

The mitochondrial DNA copy number (mtDNA-CN) is associated with ATP production and general mitochondrial enzyme activity, and thus could serve as a biomarker of mitochondrial efficiency. In practical applications, this is a low-cost, scalable assay that allows measuring mtDNA levels per cell (usually in peripheral blood cells) in a large number of samples. It is known that mtDNA-CN declines with age and is associated with frailty and all-cause mortality [101,102]. Here, we discuss the association between mtDNA-CN and atherosclerosis.

A recent study has shown an association between the low peripheral blood mtDNA-CN and the severity of CHD (coronary heart disease or coronary atherosclerosis) and increased risks for CHD [103]. Similarly, an evaluation of the association between low mtDNA-CN and PAD (peripheral arterial disease, the most common occurring in leg blood vessels) showed that lower levels of mtDNA-CN resulted in a two-fold higher risk. However, normalized scores were not significant for the mtDNA-PAD pair, while low mtDNA-CN was still linked with all-cause-mortality and prevalent and incident CVD in PAD patients [104]. Other studies have found a strong correlation between the low mtDNA-CN and SCD (sudden cardiac death) and CVD [105,106,107].

Thus, mtDNA-CN could be used as a biomarker to evaluate the risk of CVD development and particular CVD complications. Such biomarkers need to be adjusted for an individual’s variability over time, and also need to account for the difference between different populations and ethnic groups.

## 5. Conclusions

mtDNA mutations are common among CVD patients. Many CAD and hypertension-related mtDNA mutations have been reported. On the other hand, only a limited number of mtDNA mutations associated with Brugada syndrome and ischemic stroke are known so far. Available data from different studies (cybrid, cellular and animal models) suggest that the described mtDNA mutations affect the mtDNA copy number, general mitochondrial functions, increase ROS generation, decrease the efficiency of mitochondrial protein synthesis and energy production and interrupt normal Ca^2+^ metabolism and signalling.

The rapid development of sequencing technologies and accumulated knowledge about population-specific and unique mtDNA mutations help to predict the likelihood of disease development in ethnic- and family-specific ways. The investigation of every mtDNA mutation and genome individually would allow us to explore their effects on the metabolism. Respectively, this knowledge could be used for disease-preventing intervention as soon as necessary or the development and administration of personalized treatment. Similarly, the early diagnosis and treatment of mtDNA-mediated CVD would help to reduce the damage of target organs and consequences of the disease, and thus increase the quality of patients’ lives.

CVD represent a complex type of pathology that usually involves nuclear gene interactions, maternal inheritance and environmental factors. Further exploration and investigation of the underlying molecular mechanisms of every mtDNA mutation would help to create cheaper diagnostic tools, more effective treatment and define the effect of traditional risk factors (lifestyle, diet, accompanying illnesses and others).

In total, the genotyping of mtDNA mutations to determine and predict CVD could be a powerful tool for family screening and diagnosis. However, the wide application and successful use of mtDNA testing in clinical practice depend on a standardized approach to interpret mtDNA variability and level of heteroplasmy.

## Figures and Tables

**Table 1:** List of mtDNA mutations associated with CAD.

Mutation	Gene	Other Notes		References
Mt5568 (A > G)	tRNA^Trp^	Iranian CAD Patients		[34]
Mt5711 (T > A)	tRNA^Asn^			
Mt5725 (T > G)	tRNA^Asn^			
Mt12308 (A > G)	tRNA^Leu (CUN)^			
Mt16089 (T > C)	D-loop	TG	Association with CVD risk factors in Chinese Han CAD patients	[35]
Mt16145 (G > A)	D-loop	TG; LVEF		
Mt16089 (T > C)	D-loop	PC		
Mt14178 (T > C)	MT-ND6	TC		
Mt215 (A > G)	D-loop	LDLC		
Mt8231 (C > A)	MT-CO2	Iranian CAD Patients		[36]
Mt8376 (T > A)	MT-ATP8			
Mt15928 (G > A)	tRNA^Thr^			
Mt5628 (T > C)	tRNA^Ala^	Chinese CAD patients		[37]
Mt681 (T > C)	12S rRNA			
Mt5592 (A > G)	tRNA^Ala^			
mtDNA4977 Deletion	Alone or in combination with LTL associated with recurrent MACEs and all-cause mortality in Caucasian CAD patients			[38]
	Associated with MACEs and all-cause mortality in Italian CAD patients			[39]
	In combination with low folate level associated with high CAD risk among Chinese diabetic patients			[40]
Mt15910 (C > T)	tRNA^Thr^	Han Chinese patients withLHON, signs of maternally inherited CHD		[41]

## Data Availability

Not applicable.

## Abbreviations

AAA	abdominal aortic aneurysm
ATP	adenosine triphosphate
BrS	Brugada syndrome
CAD	coronary artery disease
CHD	coronary heart disease
CVD	cardiovascular diseases
ECs	endothelial cells
EH	essential hypertension
ER	endoplasmic reticulum
GWAS	genome-wide association study
HTN	hypertension
IS	ischemic stroke
LDL	low-density lipoprotein
LDLC	low-density lipoprotein cholesterol
LHON	Leber hereditary optic neuropathy
LTL	leucocyte telomere length
LVEF	left ventricular ejection fraction
MACEs	major adverse cardiovascular events
MIEH	maternally inherited essential hypertension
mtDNA	mitochondrial DNA
mtDNA-CN	mitochondrial DNA copy number
NADH	nicotine amide adenine dinucleotide
OXPHOS	oxidative phosphorylation complexes
PAD	peripheral arterial disease
ROS	reactive oxygen species
rRNAs	ribosomal RNAs
SCD	sudden cardiac death
TC	total cholesterol
TG	triglyceride
tRNAs	transport RNAs
UPR	unfolded protein response
VSMC	vascular smooth muscle cells
